# Triple contagion: a two-fears epidemic model

**DOI:** 10.1098/rsif.2021.0186

**Published:** 2021-08-04

**Authors:** Joshua M. Epstein, Erez Hatna, Jennifer Crodelle

**Affiliations:** ^1^ Department of Epidemiology, School of Global Public Health, New York University, New York, NY, USA; ^2^ Department of Mathematics, Middlebury College, Middlebury, VT, USA

**Keywords:** epidemic modelling, behaviour, dynamical systems, neuroscience

## Abstract

We present a differential equations model in which contagious disease transmission is affected by contagious fear of the disease and contagious fear of the control, in this case vaccine. The three contagions are coupled. The two fears evolve and interact in ways that shape distancing behaviour, vaccine uptake, and their relaxation. These behavioural dynamics in turn can amplify or suppress disease transmission, which feeds back to affect behaviour. The model reveals several coupled contagion mechanisms for multiple epidemic waves. Methodologically, the paper advances infectious disease modelling by including human behavioural adaptation, drawing on the neuroscience of fear learning, extinction and transmission.

## 1. Introduction

In classical mathematical epidemiology—the venerable tradition of the 1927 Kermack–McKendrick model—individuals do not adapt their contact behaviour during epidemics [1,2]. Specifically, they do not endogenously engage in social distancing based on fear. Yet, such behaviour is well-documented in true epidemics. In 2008, Epstein *et al.* published ‘Coupled contagion dynamics of fear and disease’ [3], a model that introduced the idea of two interacting contagions: one physical (the disease proper) and one cognitive (fear of the disease). Centrally, fear of disease can propagate independent of disease prevalence. The model’s core narrative is that epidemic growth induces fear. Contagious fear among healthy susceptible people, in turn, induces self-isolation. By depriving the epidemic of fuel, in the form of susceptibles, this self-isolation suppresses the disease. When disease prevalence becomes low, however, so does the fear. Thus, susceptible people (no longer fearful) come out of hiding. But, because there are still infectious individuals in circulation, this pours gasoline (susceptible individuals) on the remaining embers (the infectives), igniting a second wave. This occurred historically, in the 1918 influenza pandemic (see [4]), and history repeated itself in the multi-wave COVID-19 pandemic [5]. Recent work on the neuroscience of fear lends scientific support to the postulate of fear contagion, and a recent agent-based model explicitly includes fear modules grounded in that neuroscience; see [6,7].

In the present work, we modify and extend the original coupled contagion model [3] in light of recent advances, subsuming it in a more general framework that—while including contagious fear of disease—adds contagious fear of vaccine. The World Health Organization recently included vaccine refusal in the top ten threats to global health [8]. It is responsible for the resurgence of several deadly vaccine-preventable diseases, including measles and pertussis in the USA and even polio in several countries [9,10]. During the swine flu pandemic of 2009, roughly 40 per cent of Americans refused the vaccine [11]. And, writing as COVID-19 vaccination is underway, there is concern that refusal will undermine the attainment and maintenance of herd immunity to the SARS-CoV-2 virus and its variants.

### 1.1. The core idea

In our model, as discussed in [12], ‘Everything turns on the relationship between the two fears, one of disease, the other of vaccine.’ If fear of the disease exceeds fear of the vaccine in the population, the rate of vaccine acceptance rises, and the disease may be suppressed. However, if the prevalence of the disease is suppressed enough, fear of the disease may fall below fear of the vaccine (as might happen when a disease recedes from our collective memory). Now the vaccine is scarier than the disease, people eschew the vaccine, and a new disease cycle can explode.

This narrative also rings true historically. Smallpox, one of the great scourges of human history, kills roughly 30 per cent of those infected [13]. Yet, even when inoculation (with cowpox) was discovered, cycles of vigilance and complacency kept smallpox alive. In her social history of smallpox, *The Speckled Monster*, Jennifer Lee Carrell [14] recounts, ‘In London, inoculation’s popularity waxed and waned through the 1730s, with the force of the disease: in bad years, people flocked to be inoculated; in lighter years, the practice shrank. Inoculation was a security—the only security—to cling to within the terror of an epidemic; in times of good health, however, it looked like a foolish flirtation with danger.’ Our two-fear model generates such cycles and related dynamics.

### 1.2. Irrational epidemics: background on behavioural adaptation

The modelling literature on behavioural adaptation in epidemics has grown in several important directions; see [15–18]. Primarily, it posits that agents receive information on disease prevalence and adapt their behaviour in response [19,20]. They respond to information, not to the fears of others, as they do in our model. In the archetypal ‘rational epidemics’ tradition, agents maximize an explicit utility function, as in microeconomics and game theory, conditional on the disease’s prevalence [21–25]. While mathematically elegant, and illuminating in several important settings, prevalence-elastic optimal adaptation in the rational choice tradition is not well suited to capture prevalence independent fear contagions—‘irrational epidemics’, as it were. These come in several varieties. An extreme form is exemplified by Morgellon’s disease, an internet-disseminated delusional parasitosis [26]. For a compendious review of mass sociogenic illnesses from the middle ages to the present, see [27]. More directly relevant examples of prevalence independent fear contagion would include mass panics, such as occurred in Surat, India in 1994 [28–30], or during Ebola [31], or in recent episodes of vaccine refusal [32]. Indeed, cognitive neuroscience demonstrates that the human fear response, and fear learning generally, is not fundamentally choice-like (much less rational), or even necessarily conscious, none of which means it cannot be modelled, or estimated empirically, or counteracted [6,7,33–37]. Finally, it is also worth distinguishing our fear contagion model from those that (a) posit the conscious imitation of observable protective actions (e.g. over networks) as the behaviour transmission mechanism, or (b) that study the effect of behaviour change (often instituted as top-down policy), but not its emergence, through cognitive-emotional drivers like fear. See [4,38–42]. On the non-conscious acquisition and transmission of fear, see the discussion below and [6,7,33].

### 1.3. Organization

Regarding organization, we first present the full model (§2). Then we study a progression of four base scenarios, discussing their dynamics (§3). Analytical results and extensive sensitivity analyses are provided and discussed in §4.

We begin with the pure compartmental susceptible–infected–recovered (SIR) version of a contagious disease alone. Every subsequent scenario subsumes the preceding one, as follows:

Scenario 1: contagious disease
Scenario 2: contagious disease + fear of the disease
Scenario 3: contagious disease + fear of the disease + vaccination
Scenario 4: contagious disease + fear of the disease + vaccinations + fear of the vaccinations

These four scenarios are of central concern to public health. All numerical assumptions (parameter settings and initial conditions) are given in appendix A, ensuring replicability. Several mathematical conditions for growth are derived there as well. Appendix A also includes a pure fear ‘Salem Witches’ scenario, where fear propagates in the absence of any disease, further distinguishing the approach from prevalence elastic rational adaptation. On emotional contagion and its mechanisms, see [6,43].

## 2. The model

We first define all state variables and parameters of the model in tables 1 and 2, respectively. We use an average infectious period of 7 days (1/*γ* = 7) and a basic reproduction number (*R*_0_) of two (*β*/*γ* = 2) for the scenarios discussed.

**Table 1.** State variable definitions.

variable	description
*S*(*t*)	the proportion of susceptible individuals with no fear
*S*_fd_(*t*)	the proportion of susceptible individuals who fear the disease
*S*_fv_(*t*)	the proportion of susceptible individuals who fear the vaccine
*I*(*t*)	the proportion of (pathogen) infectious individuals
*R*_nat_(*t*)	the proportion of recovered individuals (persons who had the disease and gained immunity)
*R*_vac_(*t*)	the proportion vaccinated individuals
*A*(*t*)	the proportion of recently vaccinated individuals who fear the vaccine because of an adverse reaction
*v*(*t*)	the rate of vaccination (1/days)

**Table 2.** Parameter identifications.

parameter	description
*β*	the effective contact rate for the pathogen (1/days)
*β*_fd_	the effective contact rate of fear of the disease (1/days)
*β*_fv_	the effective contact rate of vaccine fear (1/days)
*α*_*f*_	the effective contact rate of fear loss (1/days)
*γ*	the rate of disease recovery (1/days)
*γ*_*f*_	the rate of spontaneous loss of fear (1/days)
*p*	the relative risk of acquiring the disease for disease-fearful individuals
*η*	the fear difference scaling factor
*σ*	the fraction of the rate of vaccinated that experience adverse effects
*ε*	the maximum rate of vaccination (1/days)

The mathematical model relating these variables and parameters consists of the eight coupled nonlinear ordinary differential equations shown below. For expository efficiency, we use a well-mixed model. Natural extensions would include social networks [44–47] and agent-based formulations [48].

2.1 $$\begin{array}{rl} & \frac{dS}{dt}=-\beta IS-\beta_{\mathrm{fd}}(S_{\mathrm{fd}}+I)S-\beta_{\mathrm{fv}}(S_{\mathrm{fv}}+A)S\\ & \;\;\;+\gamma_{f}(S_{\mathrm{fd}}+S_{\mathrm{fv}})+\alpha_{\;f}(R_{\mathrm{nat}}S_{\mathrm{fd}}+R_{\mathrm{vac}}S_{\mathrm{fv}}), \end{array}$$

2.2 $$\frac{dS_{\mathrm{fd}}}{dt}=-p\beta IS_{\mathrm{fd}}-\gamma_{f}S_{\mathrm{fd}}-\alpha_{\;f}R_{\mathrm{nat}}S_{\mathrm{fd}}+\beta_{\mathrm{fd}}(S_{\mathrm{fd}}+I)S-vS_{\mathrm{fd}},$$

2.3 $$\;\frac{dS_{\mathrm{fv}}}{dt}=-\beta IS_{\mathrm{fv}}-\gamma_{f}S_{\mathrm{fv}}-\alpha_{\;f}R_{\mathrm{vac}}S_{\mathrm{fv}}+\beta_{\mathrm{fv}}(S_{\mathrm{fv}}+A)S,$$

2.4 $$\;\frac{dI}{dt}=\beta IS+p\beta IS_{\mathrm{fd}}+\beta IS_{\mathrm{fv}}-\gamma I,$$

2.5 $$\frac{dR_{\mathrm{nat}}}{dt}=\gamma I,$$

2.6 $$\frac{dR_{\mathrm{vac}}}{dt}=(1-\sigma )vS_{\mathrm{fd}}+\gamma_{f}A,$$

2.7 $$\frac{dA}{dt}=\sigma vS_{\mathrm{fd}}-\gamma_{f}A,$$

and

2.8 $$\;\frac{dv}{dt}=\eta (S_{\mathrm{fd}}-S_{\mathrm{fv}})(\epsilon -v)v.$$

**Figure 1.**
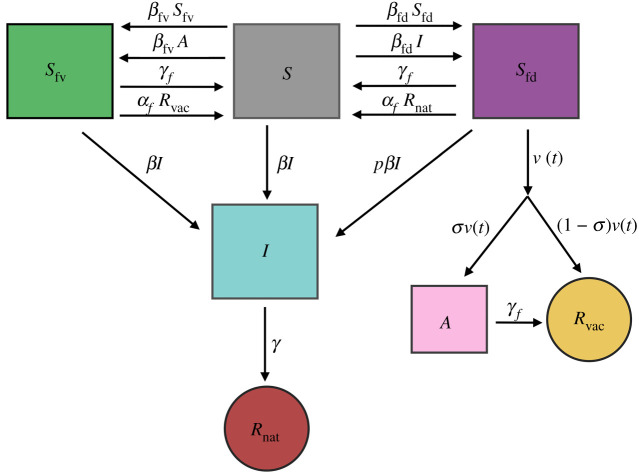
Flow diagram of equations (2.1)–(2.8).

Capital letters indicate infection states, while subscripts indicate fear states. For example, the *S*_fd_ compartment is the fraction of the population that is susceptible to the disease and fears the disease (subscript fd). The *S*_fv_ compartment is the fraction of the population that is susceptible to the disease and fears the vaccine (subscript fv). While this dynamical system is rich, as discussed below, equations (2.1), (2.4) and (2.5) reduce to the familiar SIR model when there are no fears and the terms subscripted by fd or fv are set to zero. Moreover, each of the fear contagions also propagates in classical fashion, as can be seen in equations (2.2) and (2.3). A flow diagram of the model is shown in figure 1.

### 2.1. Transmission of disease and fear

The equations include six population compartments, each representing the proportion of individuals in the given state at any time. The sum of these six compartments is always 1. A susceptible individual may acquire the disease by being exposed to an infectious person. The effective contact parameter represents the rate of transmission. When effective contact occurs, a susceptible person becomes infectious for an average period of 1/*γ* days. Once the infectious period ends, the individual recovers and gains permanent immunity to the pathogen (compartment *R*_nat_). We simplified the model by not including a pre-infectious (latent) period, although this is an obvious extension.

We consider three types of susceptible individuals: persons without fear (*S*), persons who fear the disease (*S*_fd_), and persons who fear the vaccine (*S*_fv_). A susceptible person can retain only one fear at a given time, and all fearful persons, regardless of fear intensity, are classified identically. A non-fearful (susceptible) individual may acquire fear of the disease by interacting with infectious or disease-fearful persons. These processes represent scenarios in which a susceptible person observes or communicates with an infectious (sick) individual or with a disease-fearful person. Unlike the transmission of the pathogen, such interactions could occur at a distance (as on social media) and thus require a dedicated effective contact rate parameter (*β*_fd_). Note that an infectious individual can infect a susceptible person with either the pathogen or fear of the disease, but again, not both.

### 2.2. Fear of the disease

Fear of the disease affects the behaviour of susceptible individuals. These persons may take protective actions, such as self-isolation, mask-wearing, social distancing, avoidance of travel and mass gatherings, and improved personal hygiene. In the interest of simplicity, such actions are modelled using the relative risk parameter *p*, which is used to scale down *β*. This is a fundamentally different representation than in [3], where distanced individuals were a separate compartment. Here, they are not. In the present model, a value of *p* = 0.25 represents a 75% decrease in the likelihood of a disease-fearful individual becoming infected with the disease compared to a susceptible individual with no fear. Disease-fearful individuals may also choose to gain permanent immunity through vaccination. Only individuals in this class take the vaccine, since in our model, the only motivation to get vaccinated is some level of disease fear. We assume that a small proportion (*σ*) of vaccinated individuals experience adverse effects or associate an unrelated discomfort with the vaccine. These individuals (*A*) acquire a (transmissible) fear of the vaccine while gaining full immunity. The rest of the vaccinated individuals, a proportion of 1 − *σ*, gain immunity without acquiring the fear (*R*_nat_).

### 2.3. Fear of the vaccine

Susceptible individuals acquire fear of the vaccine by interacting with vaccinated persons who had an adverse experience (*A*) or with vaccine-fearful susceptible persons (*S*_fv_). The effective contact rate of such interactions is *β*_fv_.

### 2.4. Fear conditioning and extinction

We know from neuroscience that, post-traumatic stress notwithstanding, fear is not permanent but decays in the absence of an aversive stimulus. In this model, susceptible people may naturally overcome both fears (of disease and vaccine) and join the compartment of non-fearful susceptible individuals (*S*). Our model contains two paths for such fear decay, or ‘extinction’ as it is called in behavioural neuroscience [49]. Specifically, we think of exposures (direct or indirect) to disease-infected people as classical associative fear-conditioning trials. A classic example of a fear conditioning trial is as follows. If a person is simply shown a benign blue light, no manifestations of fear (e.g. freezing, pupil dilation, adrenaline spikes, increased heart rate, electrodermal activity) or neural correlates of fear, such as activation of (e.g. oxygenation and recruitment of blood to) the amygdala, as seen in fMRI [50] are observed. By contrast, if the subject is unexpectedly given an aversive electric shock, the amygdala is immediately stimulated, triggering a suite of fear responses. Importantly, if the two stimuli are repeatedly paired—blue light followed shortly by shock—the subject will come to associate (not necessarily consciously) the light with the shock, to the point where the blue light alone elicits the same amygdala response as the shock. By a process of associative learning, the subject has been ‘conditioned’ to fear the blue light. If these light–shock pairings are discontinued, the fear of the blue light will decay. Both the fear acquisition phase and fear extinction phases can be modelled mathematically [51].

Consider a person whose fear of the disease has prompted self-isolation. This person’s fear may decay in two ways. The first is by eliminating direct (aversive) exposures to disease-infected individuals; conditioning trials are thereby suspended, and ‘fear extinction’ commences. In the model, this natural decay is exponential, consistent with the simple seminal Rescorla–Wagner model [52]. We assume that, in the absence of a fear stimulus, a person will retain fear for an average duration of 1/*γ*_*f*_ days. On the widespread use of the Rescorla–Wagner model, see [53]. For other learning models, see for example [54,55].

The second path to overcoming fear is social and distinct from extinction through stimulus deprivation. Individuals may lose fear by communicating with persons who have recovered from the fearful event. These reassuring exposures (think of repeated blue light and candy pairings) can damp the conditioned fear. This would be called counter-conditioning, over-writing a negative response with a positive one. On the relative effectiveness of extinction and counter-conditioning in diminishing fear in children, see [56]. By interacting with a recovered person (*R*_nat_), a disease-fearful person (*S*_fd_) may lose their fear. Similarly, a vaccine-fearful person (*S*_fv_) may lose their fear by interacting with a protected vaccinated person (*R*_vac_).

In our model, vaccinated persons who had gained fear due to a negative vaccine experience (*A*) abandon the fear only via the first path: natural exponential decay. We assume that their first-hand experience with the vaccine makes them resistant to social influence. Analogous to disease fear, they retain their fear of vaccine for an average duration of 1/*γ*_*f*_ days and then join the compartment of vaccinated individuals (*R*_vac_).

Widespread distancing and vaccination also cut the disease’s growth rate and can even make it negative—the herd immunity condition—which amplifies their suppressive effects.

### 2.5. Vaccine uptake

The daily rate at which fearful disease-susceptible persons vaccinate, *v*(*t*), may change over time due to a mechanism of social influence; see equation (2.8). Specifically, we assume that the growth rate of *v*(*t*) increases (d*v*/d*t* > 0) when the population prevalence of disease fear exceeds that of vaccine fear. It decreases (d*v*/d*t* < 0) when the reverse obtains—when vaccine fear is more prevalent than disease fear. We represent this effect using the difference between the two fear prevalences (*S*_fd_ − *S*_fv_):

$$\frac{dv}{dt}=\eta (S_{\mathrm{fd}}-S_{\mathrm{fv}})(\epsilon -v)v.$$

Clearly, *S*_fd_ − *S*_fv_ = 0 is a tipping point of the dynamics. Several mechanisms can affect the fear ordering. If the model begins with disease fear exceeding vaccine fear (*S*_fd_ − *S*_fv_ > 0), vaccination expands. However, this itself can endogenously suppress the disease to the point where fear of disease falls below fear of vaccine. At this point, the fear ordering switches, reversing the sign of d*v*/d*t*, opening the door for disease resurgence through vaccine refusal. Of course, two other mechanisms can drive fear of vaccine to exceed fear of disease. One is an accumulation of adverse vaccine events represented by the *A* compartment. Another mechanism (not included here) would be exogenous suppression of disease fear (*S*_fd_) through statements by officials underestimating the threat.

We turn now to the core scenarios of the model. Again, all numerical assumptions are provided in the text or appendix A.

## 3. Results

### 3.1. Base scenarios

#### 3.1.1. Scenario 1: contagious disease only

**Figure 2.**
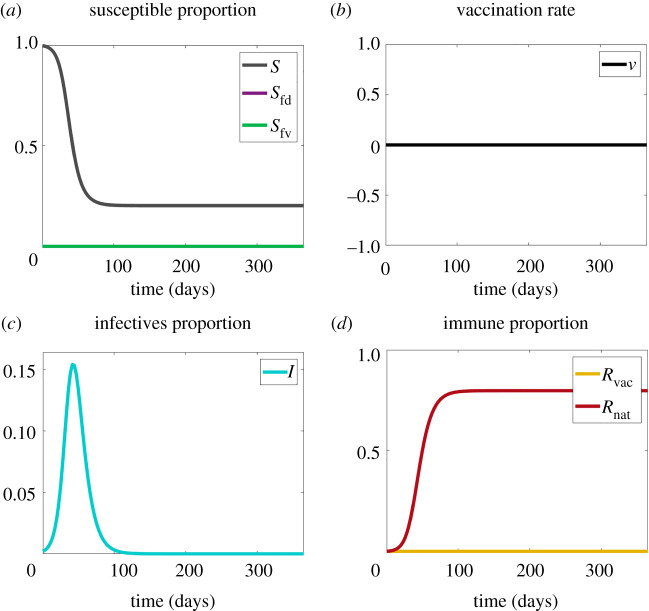
Plots for Scenario 1 (contagious disease only). (*a*) The proportions of susceptibles without fear (*S*), disease-fearful susceptibles (*S*_fd_) and vaccine-fearful susceptibles (*S*_fv_). (*b*) Vaccination rate (*v*). (*c*) The proportion of infectives (*I*). (*d*) The proportion of recovered (*R*_nat_) and vaccinated (*R*_vac_) individuals. Note that about 80% of the population become infected with the disease.

Here, we ‘dock’ the model to the classic case, an SIR epidemic with no fears, with a disease transmission rate *β*, and a single recovery (and subsequently immune) rate, *γ*. In this case, equations (2.1), (2.4) and (2.5) reduce to the Kermack–McKendrick model. A reference plot of the main dynamics is given in figure 2, which illustrates our graphical strategy. To reduce clutter, it will prove useful to have four plots focused on different aspects of the coupled contagions: susceptibles, vaccine uptake, infection and removals, as shown in figure 2.

With all fears and all vaccinations clamped at zero (figure 2*a*,*b*), we see the classical blue single peaked curve of infectives in figure 2*c*, the falling susceptible curve in figure 2*a*, and the rising recovered curve in figure 2*d*.

#### 3.1.2. Scenario 2: contagious disease + fear of disease

**Figure 3.**
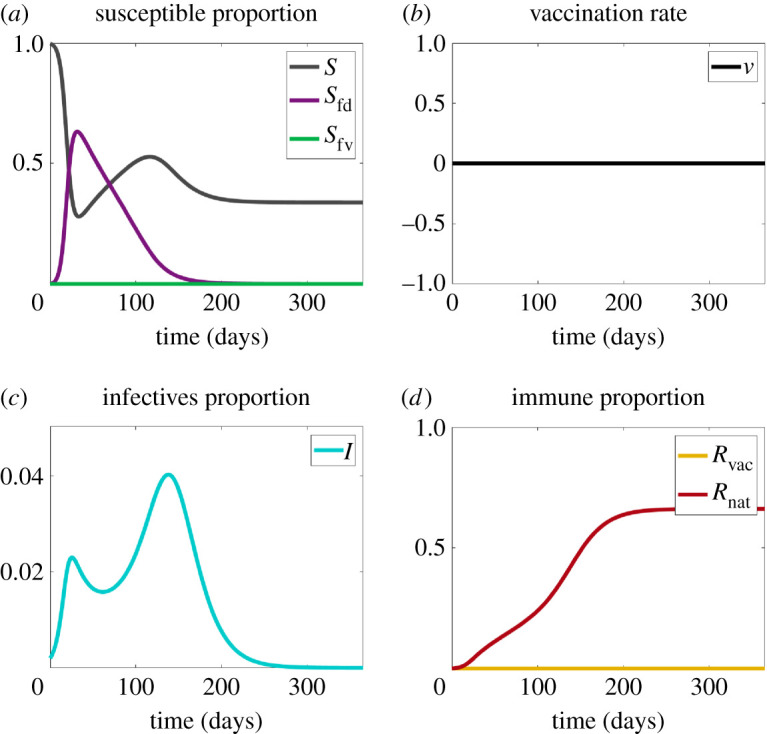
Plots for Scenario 2 (contagious disease + fear of the disease). (*a*) The proportions of susceptibles without fear (*S*), disease-fearful susceptibles (*S*_fd_) and vaccine-fearful susceptibles (*S*_fv_). (*b*) Vaccination rate (*v*). (*c*) The proportion of infectives (*I*). (*d*) The proportion of recovered (*R*_nat_) and vaccinated (*R*_vac_) individuals. Note that about 66% of the population become infected with the disease.

Now we add contagious fear of the pathogen, so there are two contagions, as depicted in figure 3. The core narrative here is that the initial spike of infections (the blue curve) stimulates a fear spike (the purple curve). People reduce their contacts out of fear (this is modelled through *p*), which suppresses disease spread. As the disease wanes, however, so does the fear of it. Now, susceptibles go back into circulation, which pours fuel on the infective embers, and a second wave ensues. The second wave is larger than the first. Why? Because in our model, there are two mechanisms of fear decay, and they amplify one another. One mechanism is the ‘natural decay’ governed by the parameter *γ*_*f*_. The second is the ‘contagious’, fear-reversal mechanism. People who have recovered from the disease are in contact with those who are still fearful. The recovereds’ low fear is also transmitted, emboldening the fearful people in hiding to come ‘out of the basement’ when it is still unsafe. This ‘complacency contagion’, if you will, amplifies the natural fear decay rate to produce a very sharp fear reduction. This pours a larger number of susceptibles onto the circulating infectives than would either fear decay mechanism alone. The result is that the second wave of the disease can be larger than the first, as occurred in 1918 [57]. We illustrate in figure 3 that two peaks of infection may appear in this scenario. Its robustness is explored in §4.

As the data science of social media shows [58], fear can spread much faster and much farther than the disease itself (a good thing when it induces preventive measures). For the earlier 2008 model, an analytic expression for the *R*_0_ of fear, and conditions for fear of disease to spread faster than the disease itself, are given in [3]. The mathematics are different here and several analytical growth conditions for the present model are given in appendix A. An obvious reason for fear to outpace disease is that disease transmission requires direct physical contact while fear transmission does not. Indeed, there are two channels to acquire disease fear in our model—through contact with an infectious person (in the *I* compartment) or contact with a frightened susceptible person, in the *S*_fd_ compartment. Scared individuals—whether sick or not—remove themselves from circulation, social distancing with an effectiveness governed by the parameter *p*. This endogenously affects the contact dynamic, and thus the disease epidemic itself. Sometimes, the self-isolation is sufficient to produce herd immunity and epidemic fade-out (see §4). In other cases, because disease prevalence is low, individuals recover from fear at a rate *α*_*f*_ despite the presence of disease. This releases fresh susceptibles onto the still-circulating infectives, generating a second wave, as shown in figure 3. We now extend the model further, adding vaccination, but not yet the fear of it.

#### 3.1.3. Scenario 3: contagious disease + fear of disease + vaccinations

Vaccination can mitigate the second wave generated in Scenario 2, a beneficial result from a public health perspective. In this and the next scenario, we assume that at time zero, no one is vaccinated, but that the vaccine is fully available throughout.

**Figure 4.**
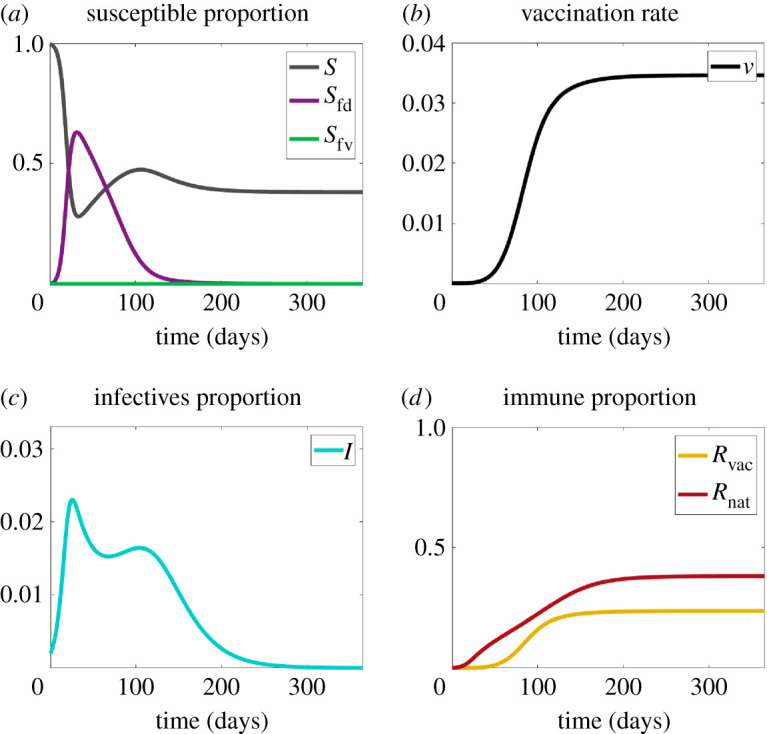
Plots for Scenario 3 (contagious disease + fear of the disease + vaccinations). (*a*) The proportions of susceptibles without fear (*S*), disease-fearful susceptibles (*S*_fd_) and vaccine-fearful susceptibles (*S*_fv_). (*b*) Vaccination rate (*v*). (*c*) The proportion of infectives (*I*). (*d*) The proportion of recovered (*R*_nat_) and vaccinated (*R*_vac_) individuals. Note that about 38% of the population become infected with the disease.

In figure 4*a*, fear of disease (purple) exceeds fear of vaccine (green), which is clamped at zero. Vaccine uptake thus increases, as shown in figure 4*b*. Now we see both ‘natural’ and vaccine-induced removals (figure 4*d*). The combined effect is to suppress the second wave, as evident from the lower-left infection curve.

Note that, while the second wave of figure 4 is clearly suppressed, there is still a small second wave. The mechanism for the two peaks here lies in the assumed effectiveness of social distancing. We have assumed that the relative risk reduction *p* of contracting the disease while fearful is low. If we increase *p*, the peaks get closer together until (in the limit) they converge to a single peak again. The sensitivity of the phenomenon to variations in *p* is given in §4 below.

#### 3.1.4. Scenario 4: contagious disease + fear of disease + vaccinations + fear of vaccinations

In Scenario 4, the fear of vaccination ‘wins’, and the outbreak is again unmitigated. People do vaccinate at the beginning of the outbreak but stop too soon because the fear ordering reverses.

**Figure 5.**
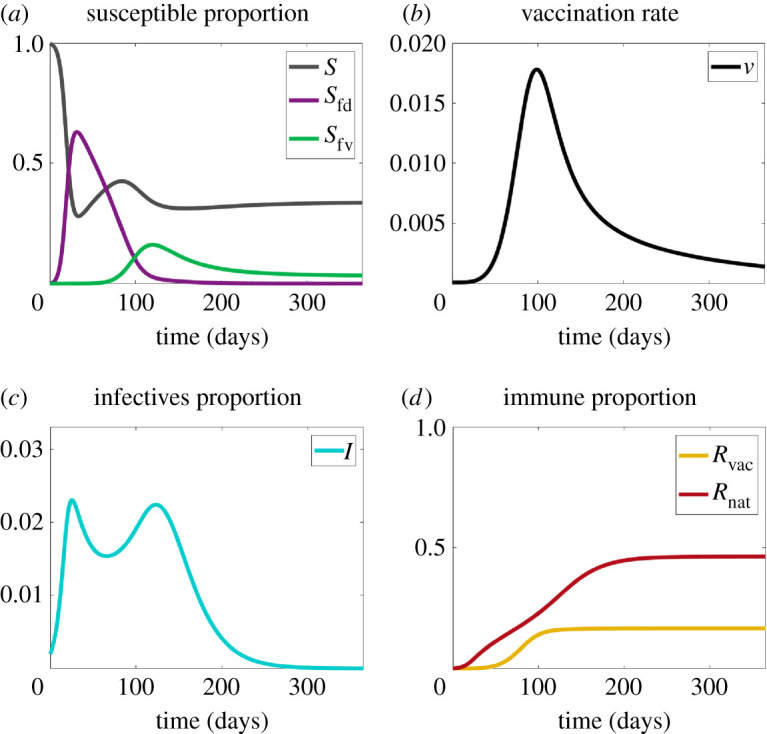
Plots for Scenario 4 (contagious disease + fear of the disease + vaccinations + fear of the vaccinations. (*a*) The proportions of susceptibles without fear (*S*), disease-fearful susceptibles (*S*_fd_), and vaccine-fearful susceptibles (*S*_fv_). (*b*) Vaccination rate (*v*). (*c*) The proportion of infectives (*I*). (*d*) The proportion of recovered (*R*_nat_) and vaccinated (*R*_vac_) individuals. Note that about 46% of the population become infected with the disease.

In figure 5*a*, we see that the fear ordering changes at roughly 100 days, at which point fear of vaccine (green) rises above the fear of disease (purple). This reverses the sign of the rate of change of *v*(*t*) (equation (2.8)), and a second wave of infections ensues.

The base scenarios exhibit several mechanisms for the emergence, timing, size and decay of multiple waves. We now explore their sensitivity to various parameters.

## 4. Sensitivity analysis

### 4.1. One fear (Scenario 2)

#### 4.1.1. Sensitivity to *p*, the relative risk reduction due to protective behaviours

**Figure 6.**
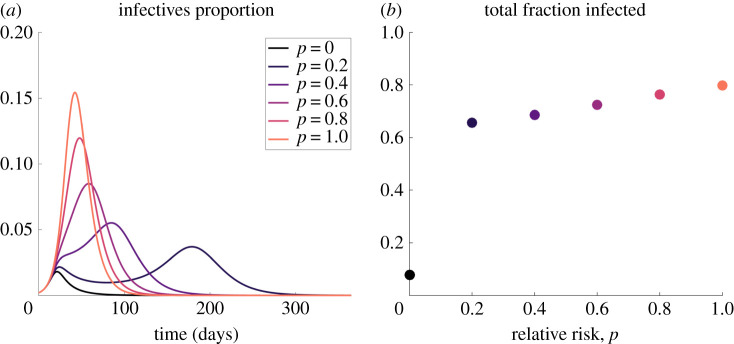
The effect of changing the relative risk, *p*, on disease spread. (*a*) The proportion of infectives (*I*) versus time. (*b*) The total fraction of the population that contracts the disease. All parameters other than *p* are as in Scenario 2 (table 3, column S2).

To begin, we return to the case of the disease and fear of the disease only (Scenario 2, §3.1.2) and study the effect of changing the relative risk *p* of acquiring the disease for disease-fearful individuals. Figure 6 shows that if *p* is decreased to 0, meaning that those who are fearful of the disease go into hiding and have a 0% chance of contracting the disease, the epidemic will be prevented (we see that only about 10% of the population gets the disease). By contrast, as *p* increases, fearful individuals become more risk-neutral (increasing their likelihood of contracting the pathogen), and the epidemic worsens; at its worst, with the fearful individuals not altering their behaviour at all (*p* = 1), we see that about 80% of the population becomes infected.

We see also that there are single-wave and two-wave regimes, depending on *p*. If *p* is small (less than about 0.4) but positive, we see a second wave emerge as fearful individuals hide away and then return to circulation. If *p* is larger than about 0.4, then the fearful individuals do not lower their risk enough to preserve a susceptible population sufficient to produce a second wave.

We note that there is a sharp bifurcation in the total fraction of the population infected for a value of *p* near 0.1 (figure 6*b*). This bifurcation corresponds to a change from one peak to two peaks, and is discussed in more depth in appendix A.

#### 4.1.2. Sensitivity to *α*_*f*_, the effective contact rate of fear loss

**Figure 7.**
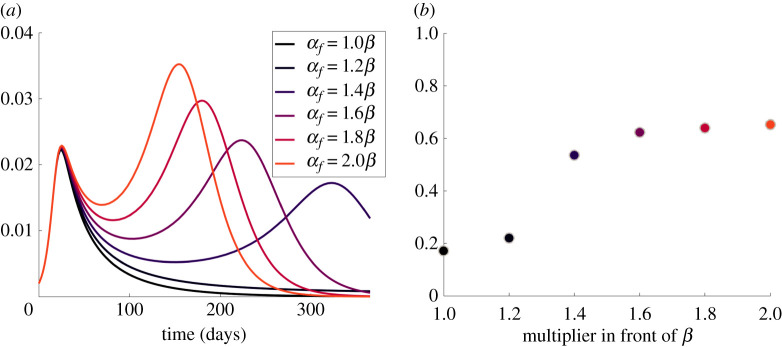
The effect of the effective contact rate of fear loss *α*_*f*_ on disease spread. (*a*) The proportion of infectives (*I*) versus time. (*b*) The total fraction of the population that contacts the disease. All parameters other than *α*_*f*_ remain as in table 3, column S2.

As noted earlier, an important extension (among several) of the original coupled contagion model [3] is our inclusion of a second mechanism of fear loss. In addition to spontaneous loss of fear, disease-fearful persons (*S*_fd_) may lose their fear by interacting with recovered persons (*R*_nat_). The effective contact rate for this interaction is *α*_*f*_. We can see that the infectives curves of different *α*_*f*_ values overlap until the first peak is reached; see figure 7*a*. The curves do not differ because the number of recovered in the beginning is too low to reduce fear significantly. The differences become apparent once the infectives curve drops. Higher values of *α*_*f*_ cause people to lose their fear and abandon their protective measures. This process increases the number of persons that are infected and in turn, the number of recovered. The larger number of recovered causes a larger fraction of persons to lose their fear of the disease and so on. The result is a second wave when the contact rate is sufficiently high. As *α*_*f*_ increases, the second peak is higher and occurs sooner. This process increases the fraction of infected persons; see figure 7*b*.

### 4.2. Two fears (Scenario 4)

#### 4.2.1. Sensitivity to *β*_fd_ and *β*_fv_, the fear contact rates

**Figure 8.**
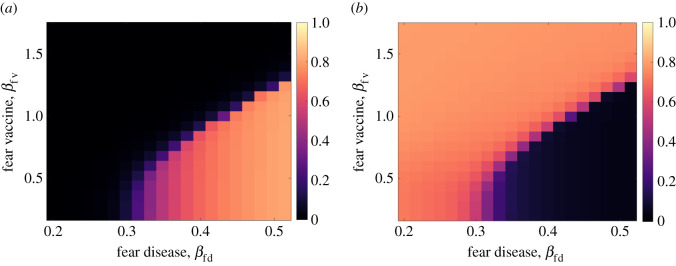
The effect of changing the fears contact rates (*β*_fd_, *β*_fv_) on the fraction of the population (*a*) vaccinated and (*b*) infected with the disease.

We now focus on the two-fears scenario (Scenario 4) and explore how the contact rates for disease fear (*β*_fd_) and vaccine fear (*β*_fv_) influence the model’s behaviour. The two effective contact rates determine how fast the fears are transmitted in the population. The fraction of vaccinated and infected persons as a function of (*β*_fd_, *β*_fv_) is shown in figure 8. When fear of the vaccine is transmitted sufficiently faster than the fear of the disease (black region in figure 8*a*), the population eschews vaccine, and a large portion of the population becomes infected with the disease (over 75%, as shown in bright orange region of figure 8*b*). When fear of the disease is transmitted fast with a sufficiently low transmission rate of vaccine fear, a small proportion of the population is infected (the black region in 8*b*).

**Figure 9.**
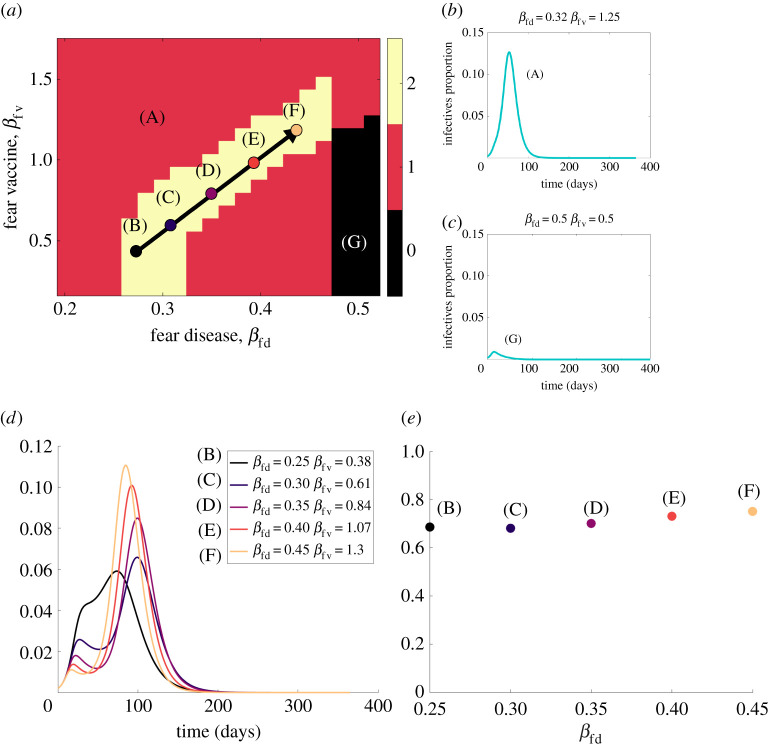
The effect of changing the fears’ contact rates (*β*_fd_, *β*_fv_). (*a*) The number of peaks in the infectives curve as a function of the two contact rates. (*b*) The infectives curve for a case of one peak. (*c*) The infectives curve for a case of no peak (no outbreak). (*d*) Five infectives curves with two peaks. (*e*) The total number of infected persons for each of the five cases.

We now explore how the fears’ contact rates (*β*_fd_, *β*_fv_) affect the number of peaks that occur in the infectives curve (*I*). We define a peak as a local maximum with a proportion of infectives above 0.01. Figure 9*a* shows that the model produces zero, one or two peaks. Point A is located in a region of fast transmission of the vaccine fear, which leads to few vaccinated individuals. Only one peak is generated (figure 9*b*) because the disease’s level of fear is too low to temporarily decrease the disease spread, and a majority of the population is infected.

Two peaks are generated in the yellow/light area of figure 9*a*. The two peaks region in (*β*_fd_, *β*_fv_) space can be well represented by the equation *β*_fv_ = 4.6(*β*_fd_ − 0.45) + 1.3 (see black arrow). Note that this area includes the interface between the orange and black regions of figure 8 which indicate the transition between the high and low fractions of vaccinated and infected persons. As we move along this arrow towards larger values of *β*_fd_ and *β*_fv_, we see that the second peak in the infection increases while the first peak decreases (figure 9*d*). As more fear enters the population, we see individuals fearful of the disease hiding out (decreasing the first peak of infection), driving the vaccination rate up, and then a disproportionate fear of the vaccine (increasing the second peak). Along this arrow, the total fraction of the population that becomes infected remains relatively constant (figure 9*e*) as the arrow is parallel to the interface of the two regions.

If *β*_fd_ is high enough (above approx. 0.45 per day), there will be no epidemic unless *β*_fv_ is sufficiently high. Even then, there will be only one peak because there are too few susceptibles to cause an initial peak high enough to reverse the order of fears (that is, too many are fearful of the disease, reducing their transmission rate); see figure 9*c*. The model does not produce more than two peaks because of the depleted susceptible pool.

#### 4.2.2. Sensitivity to *σ*, the fraction of adverse reactions

**Figure 10.**
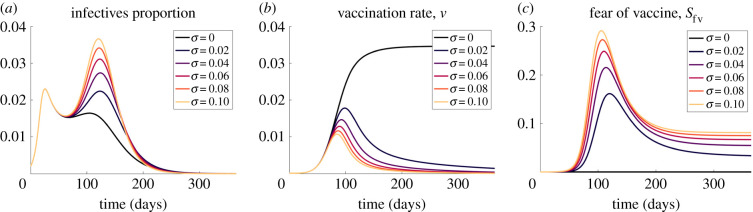
The effect of changing the fraction of adverse effects from vaccinations (*σ*) on (*a*) the proportion of infectives, (*b*) the vaccination rate and (*c*) the proportion of susceptibles that fear the vaccine.

A proportion *σ* of individuals experience adverse reactions to the vaccine and develop a temporary fear of the vaccine, which may be transmitted to susceptible individuals. As more people have such reactions, the easier it is for the vaccine fear to spread. When we increase the proportion of adverse reactions, the second peak in the proportion of infectives rises while the first peak remains unaffected (figure 10*a*). The first peak remains the same because too few persons fear the vaccine (figure 10*b*), and they have a negligible effect on the vaccination rate (figure 10*c*). After the first peak, the fear of the vaccine spreads, the vaccination rate drops, and more people become infected, leading to the second peak.

Further sensitivity analyses can, of course, be conducted. But these demonstrate how the scenario dynamics respond to variations in several key parameters. Appendix A gives parameter values and initial conditions for the scenarios and derives analytical expressions for the growth (the *R*_*n*_ values) of the disease and fear epidemics. The last of these specialize to give the condition for a fear epidemic in the absence of disease, which is also shown.

## 5. Discussion and conclusion

We have extended earlier work on coupled contagion dynamics of fear and disease [3]. In addition to a contagious disease and contagious fear of it, we have added a second contagion: fear of the control, in this case, vaccine. In addition, unlike [3], we include both the classical extinction of fear and its contagious evaporation. The interaction of these entangled contagions—of physical disease and emotion—reveals several novel behavioural mechanisms for multiple waves of infection and for their timing, size and form. Notably, these waves are generated by endogenous contagious cognitive dynamics, not by top-down policies, or by conscious maximization of utility functions, or by the imitation of observable behaviour.

Nonetheless, the triple contagion model has several limitations. As noted above, these include the assumption of perfect mixing; spatial and network variations would doubtless be illuminating. It is a compartmental model lacking diversity within the susceptible and other pools. An agent-based version could add several realistic heterogeneities. The present model is deterministic, when true epidemics are stochastic. In addition, there are certainly scenarios beyond our four that could be explored. One is the case of contagious disease and contagious anti-vaccine sentiment only. Using a different related approach, this case is studied in [59]. Finally, we do not calibrate the model to data, which will be an important empirical step.

Our broadest methodological point is that infectious disease modelling must begin to incorporate behavioural neuroscience. Human behaviour is complex and involves interacting affective, deliberative and social components. To be sure, some health decisions qualify as canonically rational. But often, as Hume noted, ‘Reason is … the slave of the passions’, the more so in settings of extreme stress like pandemics. Simple models grounded in the neuroscience of fear and its transmission can deepen epidemic modelling.

## Figures and Tables

**Figure 11.**
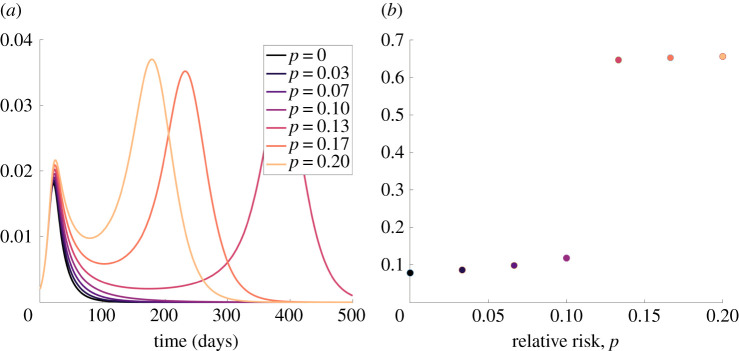
The effect of changing the relative risk, *p*, on disease spread. (*a*) The proportion of infectives (*I*) versus time. (*b*) The total fraction of the population that contracts the disease. All parameters other than *p* are as in Scenario 2 (table 3, column S2).

**Figure 12.**
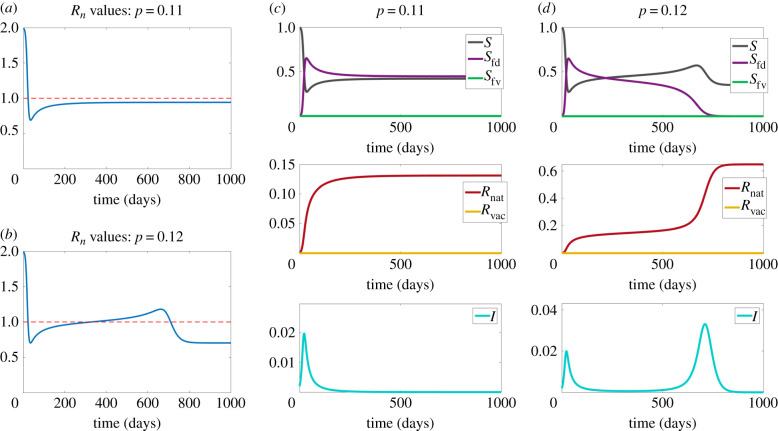
Example simulations and *R*_*n*_ values for two sample values of *p*. The reproduction number (*R*_*n*_) value for the disease plotted over time for (*a*) *p* = 0.11 and (*b*) *p* = 0.12. Simulations of the proportion of the population in each compartment for (*c*) *p* = 0.11 and (*d*) *p* = 0.12.

**Figure 13.**
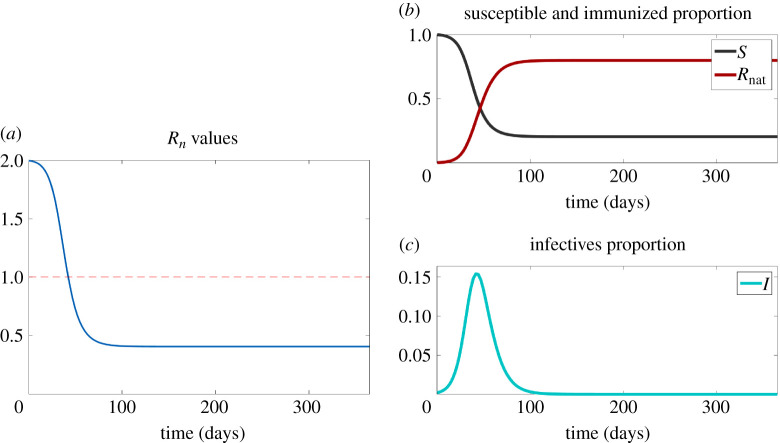
Classic SIR model (Scenario 1). (*a*) The reproduction number (*R*_*n*_) value for the no-fear case plotted over time. (*b*) The proportion of susceptible (*S*) and recovered (*R*_nat_) individuals. (*c*) The proportion of infectives (*I*).

**Figure 14.**
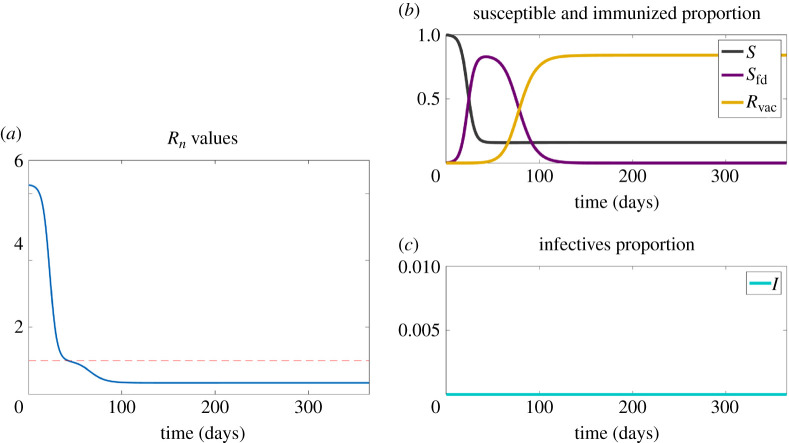
Fear epidemic. (*a*) The reproduction number (*R*_*n*_) value for the fear of the disease plotted over time. (*b*) The proportion of susceptible (*S*), disease-fearful (*S*_fd_) and vaccinated (*R*_vac_) individuals. (*c*) The proportion of infectives (*I*).

## Data accessibility

This article has no additional data.

## Appendix A. Supplementary information

### A.1. Parameters and initial conditions

The parameters employed in each scenario are shown in table 3, illustrating again the cumulative nature of the exercise. Initial conditions are given in table 4.

**Table 3.** Parameter values used in the scenarios in §3.1.

parameter	Scenario 1:	Scenario 2:	Scenario 3:	Scenario 4:
	no fear	one fear	one fear + vaccine	two fears + vaccine
*β*	2/7	2/7	2/7	2/7
*γ*	*β*/2	*β*/2	*β*/2	*β*/2
*β*_fd_	0	1.1*β*	1.1*β*	1.1*β*
*α*_*f*_	0	2.2*β*	2.2*β*	2.2*β*
*γ*_*f*_	0	0.05	0.05	0.05
*p*	0	0.25	0.25	0.25
*η*	0	0	0.8	0.8
*σ*	0	0	0.02	0.02
*ε*	0	0	0.2	0.2
*β*_fv_	0	0	0	1.6*β*

**Table 4.** Initial values used in the scenarios in §3.1.

variable	Scenario 1:	Scenario 2:	Scenario 3:	Scenario 4:
	no fear	one fear	one fear + vaccine	two fears + vaccine
*S*(0)	0.998	0.998	0.998	0.998
*S*_fd_(0)	0	0	0	0
*S*_fv_(0)	0	0	0	0
*I*(0)	0.002	0.002	0.002	0.002
*R*_nat_(0)	0	0	0	0
*R*_vac_(0)	0	0	0	0
*A*(0)	0	0	0	0
*v*(0)	0	0	0.0001	0.0001

### A.2. Growth conditions: *R*_*n*_ values

#### A.2.1. Conditions for a second peak in infections

Here, we calculate the condition for continued growth of an epidemic:

A 1  dIdt>0 βIS+pβISfd+βISfv−γI>0 β(S+pSfd+Sfvγ)>1.}

We can use this condition to understand when there may be a second peak, i.e. when *R*_*n*_ may become greater than 1 at a time point later than *t* = 0. As referenced in §4.1.1 and shown in figure 6*b*, there is a bifurcation in the proportion of infectives for low values of the relative risk parameter, *p*. If we plot the fraction of infectives for a smaller range of *p*, we see that there appears to be a bifurcation near *p* = 0.11; see figure 11

To understand this bifurcation, we look at plots of the reproduction number, *R*_*n*_, for two different values of *p*: *p* = 0.11 and *p* = 0.12; see figure 12. We note that both simulations begin with *R*_0_ = 2, and all populations, including the infectives, experience very similar trajectories. Then, after the first infection peak, we see that the fearful susceptibles, *S*_fd_, and the non-fearful susceptibles, *S*, approach similar values of about 0.45. For there to be another peak in infection, it must be the case that the reproduction number crosses 1 again. Mathematically, we require that (*β*/*γ*)(*S* + *pS*_fd_) > 1 or *S* + *pS*_fd_ > *γ*/*β*. For this simulation (Scenario 2), we have that $\frac{\gamma }{\beta }=1/2$ and we are not considering a vaccine-fearful population in this simulation (*S*_fv_ = 0). At about *t* = 200 days, right before the second peak, we estimate *S*_fd_ = *S*_fv_ = 0.45. Plugging all of this in, we have

0.45p+0.45>0.5,

yielding $p>0.11\bar{11}$. This means that for values of $p>0.11\bar{11}$, such as *p* = 0.12, the conditions of an epidemic are met again after the first peak and there will be an additional peak. If $p<0.11\bar{11}$, such as at *p* = 0.11, the condition is violated, precluding another peak. We note that once the second peak is over, the proportion of fearful susceptibles gets very close to 0, blocking a third peak.

Intuitively, the larger *p*-value above corresponds to fearful individuals taking more risks and increasing their likelihood of catching the disease. Interestingly, this model exhibits a very sharp change in disease prevalence for a small change in behaviour.

#### A.2.2. Model reduction to classic SIR

As shown in the text, in the absence of fear, our model reduces to the classical SIR model. The resulting *R*_*n*_ values are no exception. If we consider the case of no fear by setting *S*_fd_ = *S*_fv_ = 0 in equation (A 1), we arrive at the typical growth condition: *R*_*n*_ = *βS*/*γ* > 1. If we plot this value for the simulation shown in §3.1.1, figure 2, we see that when the *R*_*n*_ value is above 1, the disease is increasing; see figure 13.

#### A.2.3. Growth conditions for a fear epidemic

In a similar way, we can calculate an *R*_*n*_ value for the spread of the fear of the disease by finding a condition on which the derivative of *S*_fd_ is positive, as follows:

$$\begin{array}{rl} & \frac{dS_{\mathrm{fd}}}{dt}>0,\\ & -p\beta IS_{\mathrm{fd}}-\gamma_{f}S_{\mathrm{fd}}-\alpha_{f}R_{\mathrm{nat}}S_{\mathrm{fd}}+\beta_{\mathrm{fd}}S_{\mathrm{fd}}S+\beta_{\mathrm{fd}}IS-vS_{\mathrm{fd}}>0,\\ & \beta_{\mathrm{fd}}S_{\mathrm{fd}}S+\beta_{\mathrm{fd}}IS>S_{\mathrm{fd}}(p\beta I+\gamma_{f}+\alpha_{f}R_{\mathrm{nat}}+v),\\ & \beta_{\mathrm{fd}}\left(\frac{S+\frac{IS}{S_{\mathrm{fd}}}}{p\beta I+\gamma_{f}+\alpha_{f}R_{\mathrm{nat}}+v}\right)>1, \end{array}$$

yielding a relatively complicated condition for a fear epidemic to spread. Finally, the extreme case is fear contagion in the absence of actual disease. Baseless fear contagions are common outside public health. The Salem witch trials of 1692 come immediately to mind. But in public health there are purely psychogenic contagions like Morgellon’s disease, noted earlier. If we assume that there is no disease (*I* = 0), the growth condition above simplifies to

$$\frac{\beta_{\mathrm{fd}}S}{v+\gamma_{f}+\alpha_{f}R_{\mathrm{nat}}}>1,$$

illustrating that even if there is no infection in the population, a fear epidemic can occur. As shown in figure 14, if we begin with one baselessly fearful individual and values of disease-free *R*_*n*_ greater than 1, we see an epidemic of fear without disease, and people rushing to be vaccinated nonetheless.
